# Big Data and Comparative Effectiveness Research in Radiation Oncology: Synergy and Accelerated Discovery

**DOI:** 10.3389/fonc.2015.00274

**Published:** 2015-12-08

**Authors:** Daniel M. Trifiletti, Timothy N. Showalter

**Affiliations:** ^1^Department of Radiation Oncology, University of Virginia School of Medicine, Charlottesville, VA, USA

**Keywords:** big data, CER, effectiveness, gene, EMR, radiation

## Abstract

Several advances in large data set collection and processing have the potential to provide a wave of new insights and improvements in the use of radiation therapy for cancer treatment. The era of electronic health records, genomics, and improving information technology resources creates the opportunity to leverage these developments to create a learning healthcare system that can rapidly deliver informative clinical evidence. By merging concepts from comparative effectiveness research with the tools and analytic approaches of “big data,” it is hoped that this union will accelerate discovery, improve evidence for decision making, and increase the availability of highly relevant, personalized information. This combination offers the potential to provide data and analysis that can be leveraged for ultra-personalized medicine and high-quality, cutting-edge radiation therapy.

## Introduction

A classical tenet of evidence-based medicine is that the gold standard evidence to evaluate any intervention is a prospective, phase III randomized controlled trial (RCT) that is appropriately powered, has mature follow-up, and valid statistical analysis. This principle is emphasized particularly strongly in oncology, where the stakes are high in terms of risks of cancer mortality, morbidity from cancer treatment, and the financial toxicity of high-technology treatments.

In an ideal world, evidence from relevant RCTs would be available for each potential medical intervention with data applicable to each patient seen in the oncology clinic. However, as any clinician knows, this evidence is not available in the real world, and there are numerous challenges with this approach. The availability of an informative, practice-defining RCT requires that the researchers design the study with the end in mind: including patient selection, dosing, and other important treatment details, timing, potential therapeutic gain, number of subjects, and representation of important subgroups. RCTs often require a long time to design, conduct, and mature data, and so even well-designed studies can take decades to report meaningful results, at which time, the question asked could no longer be relevant due to other advances or trends in the diagnosis and treatment of cancer. Another significant problem with cancer RCTs is that the results of well-designed trials are commonly not applicable to patients seen in the clinic, given potential differences between patients enrolled in RCTs, who are required to meet strict eligibility criteria, and patients seen in real-world clinics, who may have a complex health history and myriad comorbidities.

Comparative effectiveness research (CER) has set out to overcome many of these weaknesses through mathematical modeling and simulation with inputs from reported scientific literature. One goal of CER is to provide evidence for medical intervention at the population level, something that clinical trials with strict inclusion and exclusion criteria could never do. Over the past decade, the validation and acceptance of CER in the US has made substantial gains (1).

Over the same time period, the collection of medical data has increased exponentially. With electronic medical records (EMR), patient databases, and genomic/proteomic data collection, we literally have more information than we know what to do with. These are examples of big data. Thankfully, many of the techniques used to store, synthesize, and interpret information of this scale have already been created and are being used commercially. NASA, the NSA, Google, Yahoo, Amazon, and Netflix, each have developed techniques to funnel oceans of information into usable packets that can predict the actions or interests of groups or single subjects (2).

Although the use of big data in healthcare research remains in its infancy, it has the potential to change the landscape of cancer care (2–5), and the integration of big data techniques and cancer therapy is an exciting arena for aspiring entrepreneurs (6, 7).

This article aims to review the future role of big data in cancer care, specifically addressing its application to CER.

## Big Data

Big data have a relatively loose definition to date, but generally it refers to amounts of information too large for human analysis. This includes data sets around 10^12^–10^18^ bytes (8). These are massive data sets; on the order of number of grains of sand on the earth (9).

Data this big is inherently heterogeneous. Consider the EMR as an example. Within a single patient’s EMR, there can be laboratory values, diagnostic reports, radiologic image sequences (every pixel), and clinical notes full of dictation errors and misspellings. In general, the information in big data sets can be grouped into structured data (numerical laboratory values or CPT codes) and unstructured data (a physician’s clinical impression text). It is not incorrect to view healthcare big data as a massive clinical database like the Farmington database. However, as discussed below, big data is hardly limited to EMR input (10).

One important difference between “traditional” research and big data-based research is that traditional research is hypothesis driven (2). This means that a research program first involves formulating a question before designing an experiment to answer that question. On the other hand, big data research may instead be data driven: those methods may first be applied to the data itself to identify potential causal relationships. This can result in a list of associations with varying degrees of correlation that can then be further evaluated. In this way, big data analysis may start the research process before identifying the important questions. Big data analytic techniques include data mining and machine learning (8), which are distinct from traditional methods of CER and offer potential alternative approaches to leveraging large data resources.

## Weaknesses

As with any modeling technique, the validity of the results is only as good as the validity of the initial data. The strength of big data is in its volume, but its weakness is in its vast heterogeneity. Data can be missing information, non-interpretable, stored in different locations, or conflicting. As one entrepreneur in cancer big data research put it, “EMR data sucks” (7). As an example, consider the inaccuracy and incompleteness of the family history in a hospital EMR. Improving the availability of big data exchanges and repositories for oncology research can only be achieved through alignment of many sectors of the health care system, which may be particularly challenging in distributed and partitioned health systems such as in the US (11). Furthermore, it is important that big data be collected and analyzed carefully to ensure that the evidence reflects the heterogeneity and complexity of the patient population (12).

Conducting big data research requires experience in several advanced analysis techniques that did not exist a decade ago, not to mention the new vocabularies that come with them (2). Realistically speaking, it is impractical for physicians to gain expertise in these techniques and they will need the assistance of public health and computer science experts, not to mention the computational hardware capable of such demanding processing and mathematical modeling (13, 14). Nonetheless, knowledge of these techniques is critical not only for researchers but also for physicians attempting to interpret the resulting publications. In order to realize the potential of a learning health system, a large number of analysts and researchers will need training in big data analytics and health care information technology (8).

Another hurdle for big data in cancer care is the ownership of the data. Although an estimated 91% of patients would permit their personal health information be shared for medical research (15), the collection and management of the data will not be performed by patients, and there will likely need to be integration of data management companies in big data research (8). Privacy is another important consideration that can limit the development of big data research in health care (11), since the potential for data breaches could harm patients and prevent health system participation in big data exchanges due to fear of data security compromises. The role that informed consent documents and institutional review boards (IRBs) should play to protect patient rights in big data research is unclear but will likely be defined in the coming years.

Because the application of big data predictive analytic methods to guide health care decisions raises concerns regarding the validity of the predictive algorithms and methods, it is important that attention be focused on validating predictive models. Clinical application of big data analytics requires trust in these methodologies on the part of clinicians, with commensurate efforts to evaluate the performance of decision support tools and information provided by big data in health care. As a result, predictive analytic models using big data to guide health care choices must be developed and executed in a transparent and replicable way with validation in real-world conditions (16).

## Integration of Big Data with CER: Opportunities for Radiation Oncology

The concept of a learning health care system focuses on continuous re-evaluation of healthcare in order to develop and apply evidence that leads to improved outcomes through a constant focus on delivering the right treatment to the right patient (8). The Institute of Medicine Report on the Learning Healthcare System recommended the development of a health care information technology infrastructure that could facilitate a learning health care system to improve health outcomes and reduce health care costs (17). The era of big data provides opportunities to leverage diverse sources of information to improve the effectiveness of radiation therapy (18). The creation of large-volume data exchanges and repositories that permit rapid pooling, synthesis, and analysis of data can accelerate discovery in radiation oncology, as well as other areas of clinical oncology (18).

Big data analytic approaches, such as machine learning and data mining, could potentially identify causal relationships between cancer therapies and clinical outcomes. CER methods can synergize with big data discoveries by providing an analytic approach to rapidly and effectively validate observations identified using big data analytics. CER methods, such as observational research studies or pragmatic trials, can be applied to the data available in exchanges and registries, or conducted prospectively through large networks in a learning health system, to validate big data observations. Since CER methods are designed to directly compare the benefits and harms of all medical alternatives, and to deliver evidence that is relevant to decision-makers, the CER framework is useful for providing data that clinicians and other stakeholders are more likely to trust than big data analytics. CER methods can be applied to create real-time decision support resources using data from large exchanges. It can also leverage the infrastructure of a learning health system to identify sites and participants for pragmatic trials to more efficiently test hypotheses (8). It should be noted that existing claims data sets and other resources used for oncology CER are relatively small compared to data sets used outside of health care research, and barely pass the threshold to be considered “big data” (8). The existing databases available for CER in radiation oncology are limited in size and data quality and have several important limitations, as described by Jagsi and colleagues (19). The availability of more and “better” data, and the synthesis of big data analytics and CER methods, would provide more opportunities for high-impact oncology research.

For radiation oncology, in particular, the combination of big data and CER has the potential to contribute to major advances toward optimizing patient outcomes. Wang has articulated a vision for the “big-data clinical trial (BCT)” (20), which may hold promise for accelerating innovation and improved outcomes for radiation oncology. BCTs would be conducted in a large population of patients, providing data that can be evaluated for insights regarding the effectiveness of therapies in subgroups, the influence of comorbidities or other factors, and even the identification of unexpected potential causal relationships using big data analytics (20). A traditional RCT could then be conducted to evaluate and potentially validate specific findings observed in a BCT (20). Alternatively, the research process could involve the opposite approach: evaluating the findings of a RCT within subgroups excluded from the trial using a BCT.

For example, one may envision potentially using BCTs to evaluate the effectiveness of radiation dose escalation, concurrent chemotherapy, androgen deprivation, or advanced technologies in different subgroups of patients receiving radiation therapy. Such a BCT would provide the opportunity for the establishment of external validity for clinical trial findings in patients otherwise excluded from RCTs or in the context of a secular trend in oncology that is hypothesized to influence outcomes. On the other hand, big data analytics such as machine learning could identify novel candidate causal relationships that could then be evaluated with CER methods such as observational cohort studies using large data exchanges. Particularly in the era of cancer genomics, one may envision leveraging large data repositories with detailed radiation therapy data and genomic profiles of tumor and normal tissue samples in order to better understand predictors of tumor control and risk of normal tissue injury, providing radiation oncologists the opportunity to potentially offer personalized dose prescriptions improving tumor control and reducing toxicity. In this way, big data could help answer important questions in radiation oncology that have been previously unanswerable.

Radiation oncology presents special challenges for the creation of large data exchanges for useful big data studies. In addition to dose–volume information from radiation therapy plans, the use of big data analytics can only be successfully applied if all relevant data elements are available. The potential list of important parameters includes diagnostic imaging to include the target volumes and proximity of normal tissue structures, detailed information from the radiation treatment plan, image-guidance data, patient comorbidities, patient demographic data, tumor staging information, concurrent chemotherapy and/or hormone therapy, detailed analyses of tumor specimens, and normal tissue samples. The relationship between diagnostic image sets (pre-treatment, during image guidance, and in follow-up) and therapeutic radiotherapy has led to the storage of a vast amount of data that could serve multiple purposes, including identifying areas at risk of disease progression or toxicity if analyzed appropriately. Big data analyses can only be useful if all potentially available data are available for analysis using machine learning or other approaches to identify potential causal relationships for further evaluation. The potential is profound, but the realization of this vision is complicated and may be too ambitious to be achieved. The currently available databases for big data and CER in radiation oncology are not adequate to support such a lofty program of ground-breaking discovery (19).

## Future Directions for Big Data and CER

Several groups have invested significant effort and money into the use of big data in cancer care (5–7). The National Institute of Health has created a “big data to knowledge” initiative (BD2K) (5), in an effort to better define and standardize the analysis of big data in health care. Additionally, there are several start-up companies forming with the aim of big data health research, some of which receive contributions from Google and other companies (7). There are multiple cohorts currently collecting data via EMR for the purpose of big data analysis (21).

Patient reported outcomes (PROs) are an ideal arena for big data research techniques and are being weighed more and more heavily as time goes on. These data sets consist of a mixture of structured and unstructured data of various utilities. Big data techniques can be utilized to synthesize these large, complex data sets into comprehensible and actionable items in cancer care (22).

Another new arena for big data research is genomic tumor analysis. The tumor genome continues to expand and the heterogeneity within tumors is extraordinary. Several commercial assays exist for breast and prostate cancer tissue analysis with many more on the way for other solid tumors. These assays utilize tissue microarrays and evaluate for genes predictive of cancer phenotype. The future of genomic analysis includes genome-wide tumor sequencing, and in some tumors it is already underway (23, 24). This would involve base pair sequencing on the order of billions.

When paired with clinical outcomes, these DNA sequences offer an ideal input for big data analysis. These results would be limited in their application if not for CER. CER could be utilized to predict the utility and cost-effectiveness of such a resource-consuming test prior to widespread adoption. This technique also has the potential to apply to whole transcriptome RNA sequencing (25), deep phenotyping (26), proteomics (27), and radiogenomics (the study of tumor genetics and their association with response to radiotherapy) (28). Future assays will likely have the ability to incorporate normal patient and tumor DNA, RNA, protein, and phenotype into a single assay that could be analyzed via big data and predictive of utility through CER. The overall trend toward increasing use of genomic assays in oncology may in fact support the development of big data resources, since the storage and analysis of genomic information requires information technology infrastructure for the secure handling of massive amounts of data.

Perhaps the most exciting possibility for big data and CER in cancer care is the idea of a “rapid learning health system” (3). This idea involves the rapid and real-time analysis of various decisions and their respective utility for a single patient. As an example, consider a middle-aged man with low-risk prostate cancer weighing his options: active surveillance, radical prostatectomy, brachytherapy, or external beam radiotherapy. The rapid learning health system has the potential to analyze his EMR and compare his health history and preferences to other patients in the data set who have chosen each of the options and offer an optimized therapy choice for that patient based on his preferences and his expected risks and gains. Biopsy genomic analysis could also be incorporated to define patient-specific tumor control probabilities for each therapy, and incorporate these data into the optimization. Surgeon or radiation oncologist-specific factors could be weighted accordingly based on who was performing the procedure. This would provide real-time, real-life decision analysis for individualized patients: ultra-personalized medicine.

One example of the application of a big data system to facilitate cancer research is North Carolina’s Integrated Cancer Information and Surveillance System (ICISS) (12). This ICISS was developed with state-level support to serve the mission of improving outcomes through a learning health system focused on improving outcomes for cancer patients. The ICISS researchers have developed a population-based data set that can be queried to evaluate outcomes, with the appropriate information technology support and environment to facilitate research to improved cancer outcomes. Details of the ICISS may be used as an example for other organizations, considering implementing similar large-scale programs aimed at creating a big data infrastructure to improve outcomes for cancer patients (http://iciss.unc.edu) (12). In addition, other large health data networks outside of oncology, such as the National Patient-Centered Clinical Research Network and the National Institute of Health’s Health Care Systems Research Collaboratory Distributed Research Network, can be used as roadmaps for developing the information technology systems to foster big data research in radiation oncology (21). The development of an information technology infrastructure to support secure data exchange and foster high-impact cancer research should be considered a priority in the movement toward realizing the vision of an effective learning health system (3).

In the current era of cost-conscious medicine and third-party payers, will big data research carry enough weight to justify payment for an intervention over the *status quo*? This is a question that is unlikely to be answered immediately or within the next 5 years, but there is much enthusiasm and potential in this line of inquiry. As big data research expands into cancer care, it will be deeply rooted in money and politics (29), and will serve as an opportunity for start-up companies and entrepreneurs (7). Regardless, the addition of CER to big data will serve to project long-term outcomes and costs for patients with cancer, which will be of value in discussion with payers. And along these same lines, it will be important to the overall health system that researchers remember to critically evaluate big data and CER findings in order to establish that the information provided by big data and CER is valuable and helpful to patients and providers (29).

## Author Contributions

Both authors contributed equally to the following authorship roles: conception and design of the work, drafting and revising, final approval of the version to be published, and agreement to be accountable for all aspects of the work.

## Conflict of Interest Statement

The authors declare that the research was conducted in the absence of any commercial or financial relationships that could be construed as a potential conflict of interest.
